# Thermal Energy Produced by Medium Velocity Pistol Projectiles and the Effects on Peripheral Nerve Tissue

**DOI:** 10.51894/001c.6345

**Published:** 2017-08-24

**Authors:** Alexander J. Colen, Logan F. Hanson, Germaine R. Frits, Cameron G. Hanson

**Affiliations:** 1 Beaumont Health Orthopedic Resident, PGY 4, Farmington Hills, MI; 2 Beaumont Health Orthopedic Resident, PGY 5, Farmington Hills, M; 3 Beaumont Health Attending Orthopedic Surgeon, Farmington Hills, MI

**Keywords:** peripheral nerve injury, thermal necrosis of nerve tissue, gunshot wounds

## Abstract

**CONTEXT:**

Sidearm pistols are more frequently involved in violent crimes due to their relatively small size and ability to be concealed. The extent to which the thermal energy released from such medium velocity pistol projectiles contributes to peripheral nerve injury requires further testing. The purpose of this paper is to describe a method to quantify how much thermal energy is released during impact of medium velocity pistol projectiles and report how thermal energy contributes to peripheral nerve injury.

**METHODS:**

Eleven seven-centimeter segments of radial, median, and ulnar nerves were dissected from a thawed fresh frozen cadaver. The nerve segments were placed in a 10% ballistics gel block, one centimeter from the end of the block nearest the shooter. A series of 115-grain 9 mm. NATO-classified ammunitions were fired through the nerve and ballistics gel construct with a pistol. The impacts were recorded with a high-speed infrared camera and nerve samples were sent for histologic analysis by two board-certified pathologists.

**RESULTS:**

The average velocity of the projectiles were 391m/s, 95% CI [387-395 m/s], with an average kinetic energy of 572.0 J, 95% CI [560.0-583.0J]. The average observable temperature of the ballistics gel/nerve prior to impact was 28.8°C±0.6ºC, 95% CI [26.4-30.3°C]. Average observable temperature of the surrounding ballistics gel/nerve during projectile impact was 55.1°C±2.4ºC, 95% CI [51.3- 62.1°C], yielding an average observable increase of 26.4°C±3.2ºC, 95% CI [20.2- 35.4°C]. An adjusted temperature increase was also surprisingly high 63.4°C ± 3.2, 95% CI [57.2 – 72.4ºC]. Histology reports of the impacted nerve tissue failed to show any sign of thermal or even crush injury.

**CONCLUSIONS:**

Medium velocity handgun projectiles release a significant amount of heat energy when impacting a substance similar to human tissue. The authors’ temperature data points were greater than those previously reported to cause thermal injury to peripheral nerves. The authors’ findings suggest that nerve injury after collision with pistol projectiles may be secondary to thermal injury in addition to the classic model of concussion and penetration given our documented levels of heat generated during impact.

## INTRODUCTION

In 2011, there were a total of over 478,400 fatal and nonfatal violent crimes committed with a firearm in the United States, leading to a total of 11,101 deaths.^1^ Between 1999 and 2008, Bartkiw et al. at the Detroit Receiving Hospital, described 2,808 reported wounds inflicted by firearm projectiles.^2^ According to US Department of Justice estimates, nine of ten violent firearm related non-fatal crimes are committed with a sidearm pistol as opposed to a rifle or shotgun.^1^ In 1994, 84% of firearm-related homicides were committed with a handgun.^1^ In 2001, 15% of state and 13% of federal penitentiary inmates carried a handgun at the time of the offense compared to 1.3% of state and 1.3% of federal inmates with rifles. Shotguns were involved in 2.4% of state and 2.0% of federal inmate crimes.^1^

It has been speculated that pistols tend to have such a higher likelihood of being involved in violent crimes due to their relatively small size and ability to be concealed.^3^ “Saturday night specials” are easily obtained illegal firearms that can be purchased for as little as $50 and found on the black-market without background checks or federal regulation.^3^ These types of pistols have often had their serial numbers removed, and may be linked to previous crimes.^3^

Several federal law enforcement branches have adopted use of 9-millimeter (mm.) NATO-classified rounds for reasons including high round capacity and relatively low user recoil. Modern day hollow point ammunition (i.e., projectiles which have a concave distal tip) expand once contacting an intended target, resulting in increased soft tissue damage and an increased level of energy transmission from the projectile to the target.

Differences in projectile velocities between categories of firearms are a major factor in tissue damage. Projectiles are categorized as “low velocity” (less than 360 meters per second m/s), “medium velocity” (370-760 m/s), and “high velocity” (greater than 760 m/s).^4^ Pistols rounds are typically in the medium to low velocity range. Two mechanisms of tissue damage from bullet impacts are shock wave cavitations causing indirect trauma via transmission of energy through the surrounding tissues (i.e., temporary cavity), and damage by direct impact and penetration (i.e., permanent cavity).^4–7^ Although larger, slower projectiles tend to damage the tissue via a direct impact crush mechanism, smaller high velocity projectiles disrupt soft tissues via shock wave stretch cavitations.^7^

Xu et al^8^ described the manner in which low-grade thermal injury to myelinated nerves results in a delayed, selective loss of myelinated fibers. Secondary heat induced angiopathy in which direct axonal damage was also seen in unmyelinated C-fibers with temperatures between 47°C and 58°C.^8^ The mechanism and severity of hyperthermic insults affecting nerve tissue has been reported in several articles^5,8,9^ However, none of these previous studies had examined the degree to which tissues are heated during impact with medium velocity pistol projectiles.

For this project, the authors hypothesized that there would be significant release of thermal energy into the surrounding tissue near the impact site of medium-velocity 115 -grain 9 mm. NATO-classified hollow point projectiles. They also hypothesized that the immediate histologic findings would fail to demonstrate gross thermal injury to study nerve segments.

## METHODS

Eleven 7-centimeter (cm.) segments of median, ulnar, or radial nerves were first dissected from a fresh frozen cadaver upper extremity immediately after being thawed. Nerve segments were placed at room temperature in a 15.2 cm. x 15.2 cm. x 40.6 cm. 10% gelatin ballistics block, one cm. from its edge closest to the shooter. The 10% ballistics gel block with embedded nerve tissues was placed three meters from the firearm.

**Figure 1:**
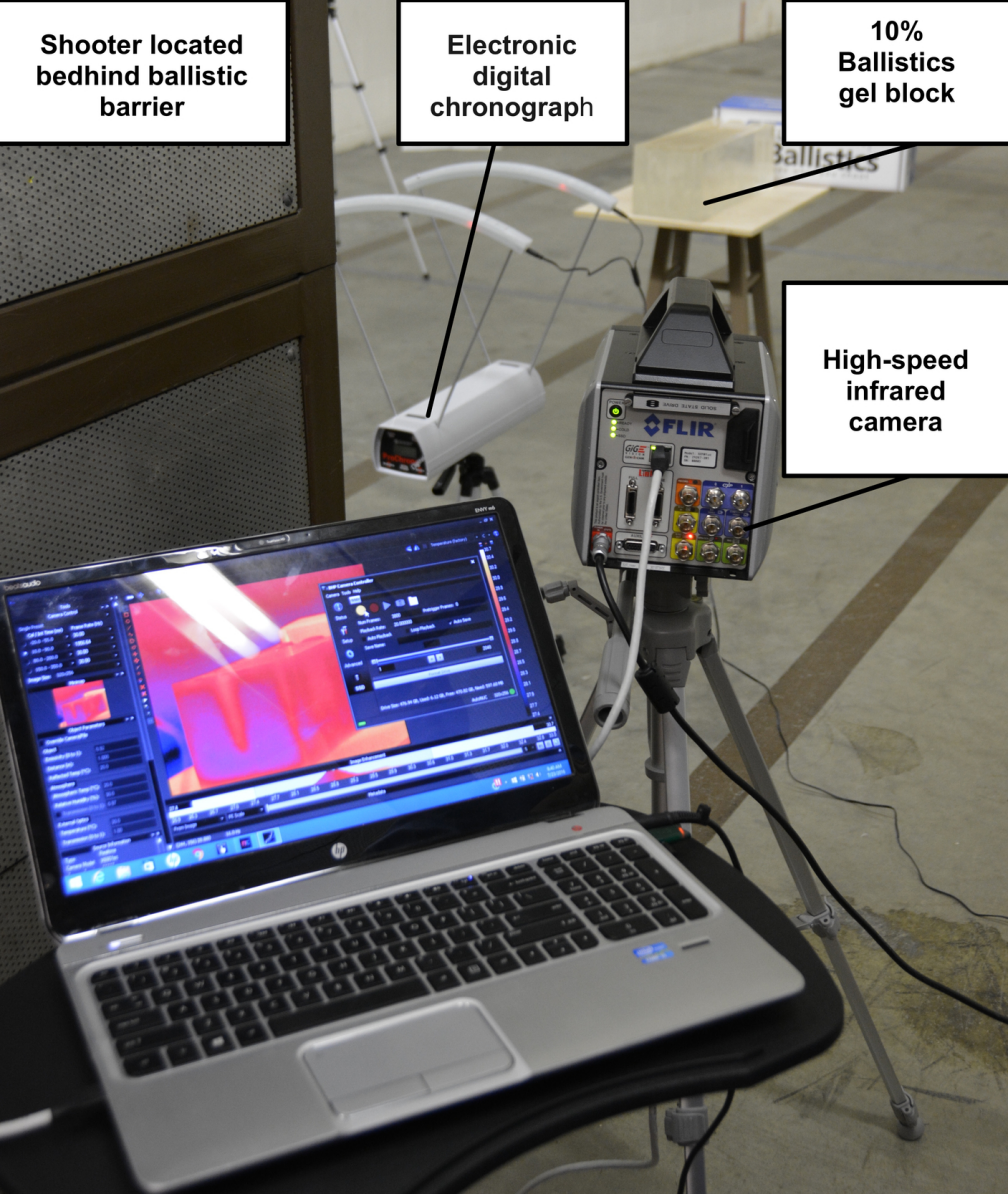
Position of IR Camera Placement Relative to the Ballistics Gel* *The shooter for this experiment was located to the left of the camera behind the ballistic shield. This setup was chosen both for the safety of the computer / camera operator and to give us access to the impact side of the ballistics gel for analysis.

An electronic digital chronograph (Competition Electronics^®^ Rockford, IL) was placed directly in front of the ballistics gel block to measure the velocities of the projectiles before impact (Figure 1). An infrared camera (IR) was also placed directly lateral to the pistol, separated by a ballistic wall for protection of the sensitive electronics and to record thermodynamics profile of the projectile prior, during, and immediately after impact. The IR camera was specifically calibrated to read temperature changes in the ballistics gel.

Standard pressure 115-grain jacketed hollow point 9 mm. NATO-classified (Underwood^®^ Sparta, IL) ammunition with an average velocity of 391.5 m/s was used for the trial. A 9 mm. 1911 pistol with an 11.2 cm. (4.4-inch) barrel was used. The pistol was fired directly at the nerve embedded in the ballistics gel, penetrating the nerve, from a distance of 3.0 meters. This distance of three meters had been chosen given that 79% of law enforcement officers who were killed with a pistol between 2006 and 2015 were at distances of 0-to-10 meters.^10^ This distance was also selected to provide an adequate distance for muzzle blast to not be a factor with our temperature readings. Five minutes were allowed between trials to allow for the ballistics gel temperature to stabilize back to room temperature as confirmed by the IR camera.

The impacted nerve tissue segments were then removed, wrapped in normal saline soaked gauze, placed in individual storage containers, and placed on ice. The nerve tissue was then sent to the Beaumont Health Farmington Hills campus histological laboratory. The samples were fixed in 10% zinc formalin for approximately 72 hours, sectioned at 5 mm. intervals and embedded in paraffin. Four-micrometer (μm) thick sections were mounted on charge slides, deparaffinized in xylene, hydrated in a graded series of alcohol and stained with hematoxylin and eosin (H&E) for histologic evaluation.

A high-speed IR camera (FLIR Systems X6901sc^®^ Wilsonville, OR) was used for the experiment. The FLIR X6901sc utilizes an Indium Antimonide (InSb) infrared detector with a broadband (3–5 μm) spectral sensitivity and F/3 aperture. The temperature range selected was from 0ºC to 208ºC ± 2ºC with a sensitivity of <20mK. The frame rate was between 1004.6 frames per second to 2000 frames per second with a resolution of 640x512. Data was recorded to the camera’s internal solid-state drive then transferred to a computer for analysis. Each recording was labeled with the active nerve trial (N1-N11) data collected during a three-second period (3004-6000 frames).

**Figure 2:**
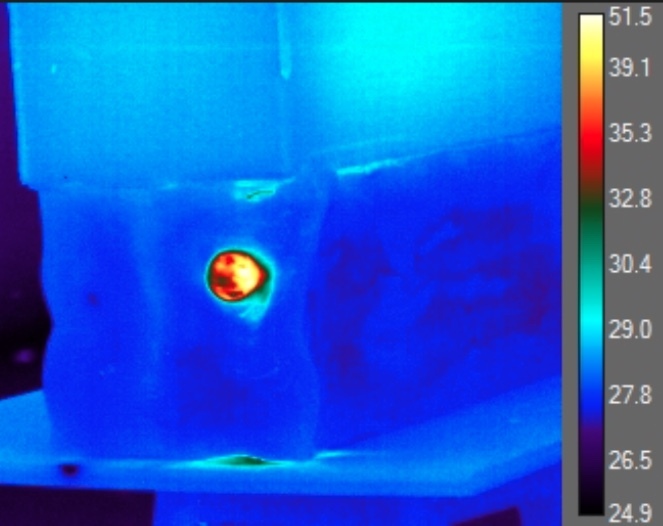
IR Signal of the Peripheral Neve Impregnated Ballistics Gel Immediately During Impact with the 9 mm. NATO-Classified Pistol Projectile* *The color scale, in relation to the designated temperature (ºC) is located to the right of the image. We used the inner most aspect of the shock wave to calculate internal temperature in º Celsius.

The IR data regarding post-impact temperatures was derived from the transverse shockwave of the impact in the gel which exposed the internal environment of the gel as well as the impacted nerve. Temperature readings were only obtained at the entry point of the gel where the nerve was located (Figure 2). After analyzing each frame of each trial, the authors had identified the maximum observable temperatures within the projectiles path along with that of the projectile itself.

The area of the maximum temperature of approximately 2-4 mm ± 1 mm was determined by using the ballistic gel dimensions as a scale reference, correcting for potential lens distortion. Each temperature reading of the impacted nerve segment and surrounding ballistics gel was sustained for approximately 2 to 3 frames, at 2000 frames per second, or 1 to 1.5 msec. The temperature of the projectile was kept consistent until impact with the ballistics gel.

Once data from the X6901sc solid-state drive was transferred to the computer, FLIR Research^®^ IR Software was used for analysis. This software allowed temperature identification for each pixel selected, in addition to temporal plots, histograms, digital counts, and radiance.

**Figure 3:**
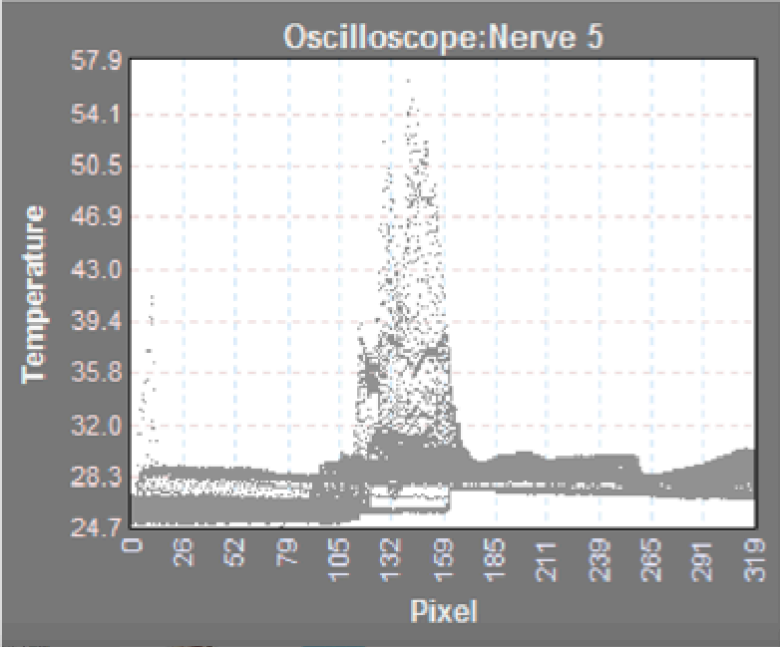
Maximum Temperatures (ºC) of the Projectile and Peripheral Nerve Impregnated Ballistics Gel Before, During and After Impact

The authors generated histogram plots concerning the areas of interest, and spatial temperature point data were used to localize the maximum temperatures of projectile and ballistics gel, and nerve prior, during, and following the impact. Each frame was individually analyzed to identify the maximum temperature throughout the projectiles path (Figure 3).

## RESULTS

Eleven nerve samples were used for this study with a length of 7 cm each. Average velocity of the 115-grain, 9 mm. NATO ammunition immediately before impact with the ballistics gel was 391.5 m/s, 95% CI [387.0-395.0 m/s] with an average kinetic energy of 572.0J, 95% CI [560.0-583.0J]. The average observable temperature of the ballistics gel immediately before impact was 28.8°C ± 0.6ºC, 95% CI [26.4-30.3°C]. The average observable temperature of the bullets before impacting the gel was 33.3°C ±1.6ºC, 95% CI [32.0-35.5°C] which was less than we originally expected. The average observable maximum temperature of the gel during impact was 55.1°C ± 2.4ºC, 95% CI [51.3- 62.1°C]. However, the average observable increase in temperature of the ballistics gel during impact was a surprisingly high 26.4°C ± 3.2ºC, 95% CI [20.2- 35.4°C] (Table 1). Our adjusted average maximum temperature was calculated to be 63.4°C ± 3.2, 95% CI [57.2 – 72.4ºC].

**Table 1:** Maximum Temperatures of Projectile Impacts*

Nerve Trial	Max Bullet Temperature (°C)	Ballistic Gel Temperature (°C)	Max Entry Temperature (°C)	Change in Temperature (°C)
Nerve 1	33.1	29.6	51.5	21.9
Nerve 2	33.5	29.7	53.8	24.1
Nerve 3	33.9	29.9	51.3	23.2
Nerve 4	32.7	26.7	62.1	35.4
Nerve 5	31.9	26.4	57.9	31.5
Nerve 6	32.0	26.4	61.0	34.6
Nerve 7	33.0	30.1	50.3	20.2
Nerve 8	32.9	30.2	53.0	22.8
Nerve 9	35.5	30.3	56.4	26.1
Nerve 10	32.4	27.8	57.5	29.7
Nerve 11	32.9	29.9	51.5	21.6

*All calculations were done with confidence interval of 0.1.

Histological evaluation of the nerve portions which had been penetrated by the projectiles were determined by gross examination prior to sectioning and could be readily identified by deformation of the normal tubular structures. On H&E evaluation, both the nerve segments that had been penetrated by the bullet and nerve tissues distal to the area of bullet impact appeared identical. In particular, there was no evidence of thermal alterations or even crush injury of the sample near or distal to the bullet impact site.

## DISCUSSION

From this project, the authors were able to observe and record how much thermal energy was released into nerve tissue embedded 1 cm. in ballistics gel during impact with medium-velocity pistol projectiles. These results bring into question how much nerve injury is caused via hyperthermic insult versus crush/penetration injury during impact of a sidearm projectile.

The peripheral nerve was embedded 1 cm. into the 10% ballistics gel to simulate in-vivo position of peripheral nerve in upper extremity as was determined by McCartney.^11^ They demonstrated that the distance from skin to median nerve throughout the upper extremity (wrist 2.1 mm ± 0.5, distal forearm 8.5 mm ±1.9, middle forearm 12.4 mm ± 2.2, proximal forearm 16.8 mm ± 2.7, and elbow 7.1 mm± 2.7) is an average of 9.4 mm.^10^

For this project, 10% ballistics gel (Clear Ballistics^®^ Fort Smith, AR) at room temperature was used for its similarity to human tissue in the manner which it transmits energy from high-speed projectiles.^7^ Increasing the starting temperature of the ballistics gel and nerve segments to normal body temperature would have altered the ballistic characteristics of the gel and would not be similar to that of human tissue.

Given that the projectile was affecting the ballistics gel and nerve as a system and not acting as a direct transferor of thermal energy from the projectile itself, the authors concluded that temperature changes would be constant regardless of the initial temperature of the gel and nerve. Given this information, the authors addressed the average maximum temperature adjustment by increasing normal body temperature of 37°C by 26.4°C to give an adjusted value of 63.4°C ± 3.2, 95% CI [57.2 – 72.4ºC].

There have been several studies describing how thermal energy may propagate nerve damage in the pertinent literature. Hoogeveen et al^12^ described how exposing peripheral nerves, utilizing an in-vivo rat model, to a heat source of 45°C for 30 minutes led to gradual decreases of motor and sensory function with complete loss to both at seven hours post heat exposure.^12^ This group reported that it had required four hours post hyperthermia insult to visualize swelling of the media, loosening of the adventitia, perivascular edema, and disruption of myelin sheaths of nerve tissue seen on histological slides.

Xu and Pollock^8^ demonstrated with an in-vivo rat model that exposing sciatic nerve tissue to low grade temperatures of 47°C correlated with a delayed and irreversible block to A-fibers (myelinated) conduction. Immediately after sciatic nerves were exposed to a heat source, there was no observable change to the nerve tissue structure under light microscopy. This is in contrast to the advanced nerve tissue degeneration which was viewed six hours post low-grade heating. One day after this low-grade hyperthermic injury, nerve fibers demonstrated damage that extended distal to the heated nerve segment.

Xu and Pollock^8^ also exposed a number of sciatic nerves to high-grade temperatures of 58°C. Immediately after the high-grade heat exposure, the myelinated fibers appeared normal overall under light microscopy. However, three days post high-grade thermal exposure, the nerve tissue demonstrated global destruction of both the myelinated and unmyelinated nerve fibers.

Lynch and Pollock^9^ described how necrosis of nerve tissue was propagated by ischemia after hyperthermic insult due to the sensitivity of the vasa nervorum to variations in temperature leading to the process of heat-induced angiopathy.

The time lag between the hyperthermic insult and progression of symptoms and histological changes in the authors’ study may explain why there was no discernable injury seen with our nerve samples post-projectile impact and hyperthermic exposure. Further research utilizing an in-vivo model, where a nerve tissue is rapidly heated 26.4°C above baseline followed by serial physical and histological examination similar to prior studies may have provided a fuller account of how nerve tissue can be injured during impact with a medium-velocity projectile.

Our findings suggest that nerve injury after pistol projectile collision may be secondary to nerve necrosis from heat induced angiopathy in addition to the classic model of concussion and penetration. More importantly, thermal injury to peripheral nerves from pistol projectiles may not be immediately apparent on physical exam as nerve necrosis from heat-induced angiopathy may be delayed.

### Limitations

One of the major limitations to this study, on a histology basis, was the use of fresh frozen cadaver nerves for our tissue samples. The use of in-vivo tissues would have been ideal for monitoring the progression of tissue destruction after hyperthermic injury. The 10% ballistics gel used was an average of 28.8°C ± 0.6º C, 95% CI [26.4-30.3°C] at baseline. Elevating the temperature of the ballistics gel might have provided a more accurate estimate of occurring temperature changes under physiological conditions. However, this would have also changed the ballistic characteristics of the gel by losing its similarity to human tissue. A second limitation of the study was that we were not able to record the length of time temperatures were maintained after impact due to limitations of our computer processing power.

## CONCLUSIONS

In summary, the authors were able to observe and record how much thermal energy was released into nerve tissue embedded 1 cm. in a substance similar to human tissue during impact with medium-velocity pistol projectiles. Our adjusted average maximum temperatures (63.4°C ± 3.2, 95% CI [57.2 – 72.4ºC]) were higher than those previously reported to cause delayed heat induced necrosis.^8,9,12^ These findings bring into question how much nerve injury is caused via hyperthermic insult versus crush/penetration injury during impact of a pistol projectile. When dealing with patients who have sustained gunshot injuries in close proximity to peripheral nerves, providers should be mindful that nerve injury may result from the generated heat during impact through a delayed process that may not be immediately observable on initial physical exam.^8,9,12^

### Conflict of Interest

The authors declare no conflict of interest.
